# Inactivation of Dairy Bacteriophages by Thermal and Chemical Treatments

**DOI:** 10.3390/v11050480

**Published:** 2019-05-25

**Authors:** Mariángeles Briggiler Marcó, Viviana B. Suárez, Andrea Quiberoni, Silvina A. Pujato

**Affiliations:** Instituto de Lactología Industrial (Universidad Nacional del Litoral—Consejo Nacional de Investigaciones Científicas y Técnicas), Facultad de Ingeniería Química, Santiago del Estero 2829, S3000AOM Santa Fe, Argentina; vivisuar@fiq.unl.edu.ar (V.B.S.); aquibe@fiq.unl.edu.ar (A.Q.); spujato@unl.edu.ar (S.A.P.)

**Keywords:** lactic acid bacteria, phage infection, dairy industry, thermal treatments, biocides

## Abstract

This article provides information on the characteristics of diverse phages of lactic acid bacteria and highlights the incidence of their presence in different dairy fermentations. As it is known, thermal treatments on raw milk and use of sanitizers in the disinfection of surfaces and equipment are strategies usually applied in dairy to prevent bacteriophage infections. In this sense, this review mainly focuses on the existing data about the resistance against thermal treatments and sanitizers usually used in the dairy industry worldwide, and the differences found among bacteriophages of diverse genera are remarked upon. Also, we provide information concerning the problems that have arisen as a consequence of the potential presence of bacteriophages in cheese whey powder and derivatives when they are added in fermented dairy product manufacturing. Finally, some important conclusions on each topic are marked and checkpoints to be considered are suggested.

## 1. Introduction

Phages that infect lactic acid bacteria (LAB) represent an important risk in dairy fermentation, as they are able to reduce product quality, or, in extreme cases, completely block cheese and fermented milk manufacture. Diverse strategies to control phages in dairy plants are used, among which physical (heating, filtration, high pressure, UV radiation, and electro-impulse treatments), chemical (biocides) or biological (strain rotation, or use of strains with improved phage resistance) ones can be cited [1]. In particular, thermal inactivation is the most common treatment applied to eliminate most microorganisms present, including those that cause spoilage and pathogens, thus assuring a good quality and longer shelf-life for the final product. Different studies have evaluated the thermal resistance of dairy phages, especially their resistance to temperatures and time traditionally used for pasteurization (LTLT: low temperature long time, 63 °C for 30 min and HTST: high temperature short time 72 °C for 15 s), temperatures employed for fermented milk manufacture (at 80 °C for 30 min or at 95 °C for 10 min), and conditions recommended by the International Dairy Federation (IDF; 90 °C for 15 min) to ensure complete phage inactivation [2]. Furthermore, biocides are used in dairies to sanitize surfaces and equipment. A sanitizer must fulfill certain acceptance criteria, such as low cost, ease of application, efficiency, antimicrobial activity, biodegradability, and not being corrosive, to be employed in industrial environments. Nevertheless, inactivation of bacteriophages is an additional selection criterion. Biocides employed in the food industry and tested against phages can be divided into two groups: those used in the laboratory and those applied in manufacturing environments. Alcohols, sodium hypochlorite, and quaternary ammonium chloride compounds are broadly employed in the laboratory, while peracetic acid and other low-cost biocides, very alkaline or very acidic, are chosen in dairy plants [3].

As is known, *Lactococcus lactis* and *Streptococcus thermophilus* are responsible for most industrial dairy fermentations, so the knowledge focusing on phages that infect these species is more extensive and deeper. Contrarily, information regarding phages attacking heterofermentative LAB of industrial importance, such as information on the *Leuconostoc* genus, can scarcely be found. Finally, even many *Lactobacillus* phages have been isolated from dairy products, for unknown reasons, these phage infections remain relatively low compared to those affecting lactococci and *St. thermophilus* [4]. This article is intended to review diverse thermal and chemical treatments applied in dairy factories for sanitation of raw material, equipment, and environments, focusing specifically on their bacteriophage inactivation efficiency.

## 2. Efficiency of Thermal and Chemical Treatments on the Inactivation of Dairy Bacteriophages

### 2.1. Lactococcus Bacteriophages

Strains of *L. lactis* are widely used in the elaboration of numerous fermented dairy products, and they are among the most economically important lactic acid bacteria (LAB). This genus plays a vital role in the manufacture of products, such as several types of cheeses, buttermilk, and sour cream [5,6]. Nowadays, commercial LAB-mediated fermentations have evolved into a multibillion-dollar industry, mostly employing defined starter cultures to satisfy individual dairy company’s needs [7].

*Lactococcus* phages are among the most studied bacterial viruses, and over 700 lactococcal phage isolates have been reported in the literature [8]. All lactococcal phages belong to the *Caudovirales* order, which are placed into one of two families according to their tail morphology: the *Siphoviridae* (long non-contractile tail—most lactococcal phages) and the *Podoviridae* (short non-contractile tail—few lactococcal phages) [7,8]. The current number of lactococcal phage groups was reduced from 12 to 10, including the emerging groups Q54 and 1706, by using DNA hybridization, restriction profile analysis, genome sequencing, and electron microscopy [6]. Most lactococcal phages isolated from commercial dairy fermentations belong to three species, namely 936-, c2- and P335-type phages [9,10,11,12,13,14]. Members of the 936 and c2 species represent lytic bacteriophages with a small isometric (morphotype B1) or prolate head (morphotype B2), respectively. Like the 936-type, P335-type phages have a small isometric head, but differ from the other two species in that members of the P335 species can either be lytic or temperate. On the other hand, many studies report that phages belonging to the 936 species are the most frequently isolated in dairies, followed by c2-like and P335-like phages [9,10,11,12,15].

#### 2.1.1. Thermal Inactivation

As known, raw milk is one of the most prevalent sources of bacteriophage contamination in dairy, and thus in the manufacturing processes of fermented products. Thermal treatments, routinely performed in dairies in order to eliminate pathogens and extend the shelf life of milk, constitute one of the main barriers against phages [16]. Knowledge about the thermal resistance of lactococcal bacteriophages has been the purpose of several studies because of the worldwide importance of *Lactococcus* strains as starters in the manufacture of diverse fermented dairy products.

As reported, thermal treatments cause morphological changes on phage particles. Electron microscopic analysis, before and after thermal treatments, were performed on two lactococcal phages, P008 and P680, both members of the 936-phage species [17]. The heat-sensitive phage P008 demonstrated extensive morphological damage in comparison to its heat-resistant counterpart, P680. Release of phage DNA from viral capsids, parting of the phage into head and tail structures, and aggregation of phage tails were the most frequently seen phenomena, mainly for the heat-sensitive phages studied. These findings indicate that the heat resistance of phage P680 is based both on the increased stability of phage DNA packaged in the phage capsids and enhanced stability of phage particles. Regarding the effect of the suspension media on the thermal resistance of both phages, this study [17] demonstrated that the media containing milk proteins (whey or casein) enhanced the resistance of both phages, while different fat levels in the media did not change the rate of phage inactivation. On the other hand, an accelerated inactivation was observed for both phages in the media composed of either salt or carbohydrate solutions.

Thermal inactivation of *L. lactis* phages has been exhaustively conducted in numerous studies, including inactivation kinetics studies (Table 1). It is well known that *L. lactis* phages can resist the usual thermal treatments used in milk sanitization, such as LTLT and HTST. In this sense, Suárez and Reinheimer [18] studied the inactivation of four Argentinian lactococcal bacteriophages at 63 °C, 72 °C and 90 °C, in three culture media, RSM (nonfat dry skim milk), a TMG buffer (10 mM of Tris-HCl, 10 mM MgSO_4_, and 0.1% w/v gelatine) and M17 broth. Results demonstrated that all phages needed more than 15 s at 72 °C to achieve 99% of inactivation. Similarly, 80% of Turkish lactococcal bacteriophages treated by heat in M17 broth showed counts >10 PFU/mL after treatment at 72 °C for 15 min [19]. These results indicated that the traditional combinations of temperature and time were not sufficient to completely inactivate (from initial concentrations of 10^9^ PFU/mL) most of the lactococcal bacteriophages in the M17 broth. In addition, Madera et al. [11] observed diverse behaviors when different species of *L. lactis* phages were treated in a plate heat exchanger at 72 °C for 15 s. They reported that 936-like phages were approximately 35 times more resistant than c2 phages, and P335-like phages were the least resistant. Similar behaviors were obtained by Müller-Merbach et al. [20] for lactococcal phages P001 and P008, belonging to c2- and 936-species, respectively, when heat inactivation assays were performed in calcium enriched-M17 broth. In fact, under conditions of short-time pasteurization (72–75 °C for 30 s), phage P001 was inactivated by 7 log orders, while phage P008 was only reduced by 2 log orders. Furthermore, a study focused on the thermal resistance of c2-like phages was performed by Marvig et al. [21]. This work assayed nine *L. lactis* c2-phages by heat treatments conducted at 65 °C, 70 °C, 75 °C, and 80 °C during 5 min, in skimmed milk. Three phages displayed high sensitivity towards heat resulting in reductions of >8 log orders after 5 min at 70 °C, whereas the most thermal resistant phages required 5 min at 80 °C to obtain the same reduction. These data clearly demonstrate a pronounced variation in thermal resistance (by 10 °C) within the nine lactococcal c2-phages screened. In contrast, Madera et al. [11] concluded that lactococcal c2-phages were inactivated within a very limited temperature range and were sensitive to pasteurization processes (72 °C—15 s). However, it is certain that lactococcal c2-phages are, in general, more sensitive to high temperatures compared with lactococcal 936 phages. This fact was reinforced with the results obtained in the study performed by Murphy et al. [22], where the thermal resistance of eleven 936-type phages was assayed. In this work the assays were carried out in RSM at 85 °C for 30 min. Four out of the eleven phages were found to be considerably thermotolerant at 85 °C. However, all phages used in this study were completely inactivated by exposition at 90 °C for 5 min.

In view of the high resistance evidenced by lactococcal phages, an extensive study was performed by Atamer et al. [15] in order to obtain an indicator or a “test phage” for adequate heat treatment of milk and milk derivatives. With this aim, 56 phages were isolated from samples of whey, cheese brine, yoghurt, sour cream, quarg, and fresh and hard cheeses, with 42 belonging to 936-, 11 to c2- and three to P335-species. Thermal treatment of 80 °C for 5 min was supported by 23 phages (41%) in UHT-skim milk and 14 (25%) in water. At the same time, four phages were also able to form plaques after hard heat treatments of 85 °C and 90 °C during 5 min, either in milk or water. When the temperature was raised to 95 °C, all phages were completely inactivated except for phages P1532 and P680, both belonging to the 936 phage species. The phage P1532 was more resistant than P680 and was still detectable after a heat treatment at 90 °C for 20 min or at 97 °C for 5 min. Since the two phages belonging to 936 are the most resistant, they could be selected as appropriate test phages to mimic the “worst case” scenarios in dairy, that is, contamination with heat-stable phage populations.

In recent years, modern dairy industries have been implementing practices of recycling whey proteins and whey fat with the purpose of increasing the yield in cheese manufacturing or enhancing the texture of fermented milks. However, the re-use of native whey preparations carries the risk of contaminating the raw materials and dairy environments with phages potentially present in these new dairy ingredients [23]. In this sense, Atamer and Hinrichs [24] studied the thermal inactivation kinetics of the highly thermo-resistant test phage (P680) in whey, whey protein concentrate (WPC), and whey cream, in a range between 70 and 90 °C. After a heat treatment at 90 °C for 15 min, only a 6 log reduction was obtained, and the phage was still detectable in each medium. The kinetic parameters obtained showed that temperature/time combinations ranging from 100 °C for 20 min to 140 °C for 2 s are necessary to inactivate high concentrations (approx. 10^9^ PFU/mL) of phage P680 in all media assayed. These conditions far exceed those normally suggested for recycled WPCs (from 75 °C for 30 min to 90 °C for 5 min) and whey cream (93 °C for at least 30 min).

In view of the demonstrated protective effect of whey proteins on phage inactivation, and considering the whey derivates as new ingredients in dairy manufacture, it is essential to know the nature of such a protective effect. In this sense, Geagea et al. [25] selected the virulent phage P1532, which is highly thermal resistant, to study the influence of WPC and the individual whey components, β-lactoglobulin (BLG), α-lactalbumin (LAC), and bovine serum albumin (BSA) under different heat treatments and pH conditions. Results indicated that WPC showed a protective effect on phage P1532, but this effect depended on pH and time of treatment at 95 °C. A combination of pH 5 and a 20 min of treatment at 95 °C could significantly reduce the risk of phage contamination by WPC. However, these conditions are quite aggressive to preserve the functional properties of whey proteins, so new alternatives to reduce times or the application of diverse physical methods should be investigated. This study verified that the protective effect of WPC is not restricted to one WPC protein, but a combination of at least three whey proteins (BLG, LAC, and BSA).

The high thermal resistance of dairy phages in general, and lactococcal phages in particular (especially those belonging to 936 species), accompanied by the practice of recycling whey derivatives in fermentative processes, illustrate the risk of using these new dairy ingredients due to their potential as a source of phages. This has become an emergent and worrying problem, which is essential to study in order to minimize it. This entire situation is well exposed in the study performed by Wagner et al. [26], in which thirteen whey powders and five whey powder formulations were screened for the presence of dairy bacteriophages. Results demonstrated that lytic *L. lactis* phages were detected in all samples (a total of 33 phages isolated), in concentrations ranging to 10–10^7^ PFU/g. Most of these phages (31) belonged to the 936 group, even with significant morphological variations related to head sizes, tail lengths, and additional tail decorations. The other two phages were classified as members of the P335 group. Additionally, these phages were studied in order to evaluate their thermal resistance and the kinetic parameters of inactivation in skim milk [27]. Ten *L. lactis* phages were still able to form plaques after 5 min at 85 °C, and four of these phages even survived a temperature of 95 °C (5 min). These four *L. lactis* phages belonged to the 936 group, known to include representatives exhibiting extreme thermal stability in milk and in whey derivatives [15,24]. The most heat-resistant *L. lactis* phage currently known (i.e., phage P680 [15]) has been previously reported to survive 5 min at 95 °C. Phage P956 was still active after a heat treatment at 97 °C for 10 min and, therefore, seems to be even more thermo-resistant than phage P680. In addition, this work clearly exposed that the percentage of phages with exceptional thermal stability isolated from these powders is higher than the percentage found in “standard” dairy products. The large amount of heat resistant phages found in whey powder clearly underlines the risk of re-utilizing phage-contaminated whey powders in the dairy industry. A critical heat treatment required to achieve a complete inactivation of phages with extraordinary heat resistance in whey powder is not practically applicable, as this would result in a pronounced deterioration of all by-products. Moreover, Wagner et al. [16] assayed the thermal inactivation of highly heat-stable phages P680, P1532, and P635 in a pilot-plant pasteurizer after suspension in raw milk (10^7^ PFU/mL). Results demonstrated that thermal treatment in a pilot-plant plate heat exchanger, continuous and working under turbulent flow, was considerably more efficient than inactivation performed in laboratory conditions [15,21]. Despite this, the conditions of industrial pasteurization of milk (72 °C for 15 s) were not enough for effective inactivation, even for the phage P008, taken as a model of heat-sensitivity.

Thermal inactivation of *L. lactis* phages has been extensively studied, evidencing a wide range of behaviors against heat treatments and virions with extraordinary thermal resistance. In this sense, the protocol used for the traditional microbiological detection of phages in dairy samples (which would allow distinguishing between phages and non-phage inhibiting agents) includes a heat treatment at 90 °C for 15 min for the samples to be analyzed [2]. However, this step of the standard protocol might not be suitable for the inactivation of highly heat-resistant phages [15]. In addition, the isolation of phages with unusual thermal resistance increases every day. Thermo-resistant phages are part of the natural phage populations in all types of dairy, and they can be isolated from various dairy products. Thus, the recent practice to use derivatives of cheese whey in subsequent fermentative processes probably produces a natural selection of them as consequence of the technological processes involved. In fact, Wagner et al. [27] proved the hypothesis that phages isolated from spray-dried whey powders and whey powder formulations have higher heat resistance than phages isolated from “standard” dairy samples.

#### 2.1.2. Chemical Inactivation

Data about the sensitivity of dairy phages against biocides are scarce compared to those related to heat resistance. Table 2 shows biocides studied for inactivation of dairy phages. Biocides investigated only for lactococcal phages are described in Table 3. Ethanol and isopropanol were tested on different *Lactococcus* phages at concentrations oscillating between 10% and 100% (v/v), but only high concentrations of ethanol produced an observable effect. In this regard, Suárez and Reinheimer [18] evaluated the inactivation ability of alcohols (ethanol and isopropanol diluted in M17) on four lactococcal phages isolated in Argentina. The results demonstrated that ethanol was more effective than isopropanol at all concentrations assayed. However, ethanol concentrations that guaranteed the complete inactivation of *Lactococcus* phages after 45 min of treatment were 100% (v/v) and 75% (v/v), while concentrations of 10% (v/v) and 50% (v/v) were not effective. In the same way, Buzrul et al. [19], evaluated different alcohols on *L. lactis* bacteriophages, obtaining similar results. Ethanol at a concentration of 75% produced the fastest inactivation, while a concentration of 50% was more effective than 100%. On the other hand, isopropanol was demonstrated again to be less effective than ethanol. In another study, Campagna et al. [28] showed that phage P008 was extremely resistant to high concentrations of ethanol and isopropanol (assayed concentrations of 70% and 80%).

Regarding the inhibitory effect of sodium hypochlorite, Suárez and Reinheimer [18] evaluated its inactivation ability (diluted in phosphate buffer 0.1 M, pH 7) and reported that concentrations of sodium chloride between 100 ppm and 300 ppm completely inactivate high phage concentrations. In turns, Murphy et al. [22] investigated eleven phages belonging to the 936-type. Nine of these phages were highly resistant to 800 ppm sodium hypochlorite (prepared in distilled water), a concentration that far exceeds industrially applied standards (200 ppm). Curiously, extreme concentrations of sodium hypochlorite (between 2000 ppm and 5000 ppm) were required to reduce the 6 and 7 log orders high initial concentrations of ten Turkish lactococcal phages when the biocide was diluted in M17 broth. These high concentrations could be due to the oxidizing effect that the sodium hypochlorite generates on the M17 broth [29].

Peracetic acid is considered to be the best practical agent for phage inactivation in the dairy industry due to its rapid action and its broad spectrum [3,30]. In this regard, Suárez and Reinheimer [18] reported that peracetic acid (0.15%, 40 °C) quickly inactivated phage suspensions (viral counts below the detection limit, at <10 PFU/mL) after 5 min of treatment [18]. Similar results were reported by Murphy et al. [22] when *Lactococcus* phages were treated with peracetic acid (0.015%, v/v, pH 3.50).

Several studies have analyzed the sensitivity of *Lactococcus* phages against different commercial sanitizers used in the industry. In this sense, Murphy et al. [22] investigated the robustness of eleven phages belonging to the 936-type for their response to biocidal and surface disinfectants. The biocides assayed were sodium hydroxide (0.2%, w/v, pH 12.64), lactic acid (300 mM, pH 2.16), 1% Virkon (active ingredient, potassium monopersulphate, 49.7%; Antec International Ltd., Rotterdam, The Netherlands), Spor-Klenz (4.5% peracetic acid, 22% hydrogen peroxide, and <10% acetic acid; Steris, OH, USA) and a 1:10 dilution of a generic surface quaternary ammonium compound (QAC) (alkyldimethyl benzyl ammonium chloride; JC products, Cork City, Ireland). All phages were rapidly inactivated by 0.2% sodium hydroxide and two surface disinfectants (Virkon and Spor-Klenz). The QAC disinfectant failed to reduce phage infectivity. All phages were susceptible to high concentrations of the major fermentation by-product, lactic acid (300 mM). These findings indicate that sanitation procedures applied in dairy industry environments have a varied and phage-specific ability to reduce phage infectivity.

Food-grade sanitizers were tested against several virulent dairy phages, including lactococcal phages P008, CB13, AF6, P1532 of the 936 group, as well as P001 (c2), Q54, and 1358 [28]. The 21 food-grade chemicals included oxidizing agents, halogenated agents, alcohols, QACs, anionic acids, iodine-based acids, and an amphoteric chemical. Phage P008 was first exposed to each sanitizer for 2 and 15 min at room temperature at two different concentrations, namely the lowest and highest no-rinse sanitizing concentrations. Organic matter (whey or milk) was also added to the testing solutions. The five most efficient sanitizers against phage P008 were then tested against all phages. Chlorine dioxide (20 and 50 ppm), anionic compound E (phosphoric acid, 10–30%; 2-hydroxypropanoic acidm 1–5%; sodium alkylnaphtalene sulfonate, 10–30%; octanoic acid, 3–7%; decanoic acid, 0.5–1.5%, at concentrations of 2000 and 5000 ppm), all iodine-based acids (25 and 50 ppm), and the amphoteric compound (alkylamino carboxymethylaminopropane, sodium salt 5–10%; 200 and 500 ppm) were inefficient against *L. lactis* phage P008; therefore, they were not further tested against other dairy phages. The oxidizing agents (peroxide and peroxyacid mixtures) and the QACs (based on benzalkonium chloride) were the most efficient against all phages. Some oxidizing agents, such as sodium percarbonate (30–60% solid; 200 and 500 ppm) and Oxi-C (peracetic acid, 15–17%; acetic acid, 33–38%; hydrogen peroxide, 9–11%), were efficient against phages at their lowest sanitizing concentration, but only after a prolonged contact time (15 min), which is likely not suitable for fast-sanitizing steps. The chemical, Oxi-D (peracetic acid, 3–7%; acetic acid, 15–40%; hydrogen peroxide, 5–10%; L-octanesulfonic acid, sodium salt, 3–7%; octanoic acid, 1–5%) was, in general, the most effective biocide against all phages and, thus, its efficiency was taken as reference.

The last systematic study about biocides action against lactococcal 936-group phages was performed by Hayes et al. [5]. This investigation assessed the effectiveness of commonly employed sanitizers in the neutralization of lactococcal phages and represents the most in-depth investigation of dairy phage resistance to sanitizers to date, involving 36 bacteriophages and 14 antimicrobial compounds. Significant variations in resistance to biocide action were evidenced among members of this important phage group. Pure compounds (benzalkonium chloride, polyvinylpyrrolidone-iodine, hydrogen peroxide, sodium percarbonate, sodium dichloroisocyanurate, and sodium chlorite) and commercial sanitizers (A–F) with diverse chemical natures were assayed. Sodium percarbonate, sodium chlorite, and sodium dichloroisocyanurate were ineffective virucidal agents against this phage collection. QAC-based biocides, such as benzalkonium chloride (pure) and Sanitizer A (C8–C18 alkyldimethyl chloride ammonium compound), were found to be the most effective disinfectants. However, Sanitizer D (also QAC-based biocide; ethanol, 10%; chlorhexidine digluconate, 10%; tetradecyl-trimethyl-ammonium-bromide, <1%) was ineffective at the concentrations examined, even beyond the values suggested by supplier. This fact demonstrated that not all QAC-based biocides are suited for dairy phage inactivation. The sodium hydroxide-based sanitizers (B and C) used in this study supported previous findings of the efficacy of sodium hydroxide as a virucidal agent. Since these sanitizers are complex mixtures of chemicals, the use of sodium hydroxide at the concentrations employed during assays would probably have little effect, but the combined action with the other components (sodium hypochlorite and others in Sanitizer C and the unknown list in Sanitizer B) resulted in highly effective phage inactivation. Regarding sanitizers E (polymeric-biguanide-hydrochloride) and F (30% nitric acid, 5% orthophosphoric acid), and even though some inconsistent results were obtained and reported by the authors, significant levels of infective phages were detected after treatments at a concentration higher than those recommended by the suppliers.

The available information reveals large variations in resistance to diverse biocides for lactococcal phages. Besides, phages resistant to biocidal activity tend to possess resistance to more than one disinfectant agent. Consequently, it is apparent that phage biocide resistance is an issue that must be carefully monitored in the dairy industry, and a regular testing of sanitizers against emerging new phages should be implemented. The application of sanitizers containing a mixture of compounds could mitigate this issue in combination with additional hurdles, such as thermal and high-pressure treatments. Additionally, as a precautionary measure to avoid selecting resistant phage population, a rotation of different sanitizers should be considered.

### 2.2. Leuconostoc Bacteriophages

Bacteria of the genus *Leuconostoc* are incorporated into dairy starter cultures due to their ability to produce important metabolites, such as diacetyl and CO_2_ [46,47]. Specifically, CO_2_ expands the mechanical openings in blue-veined cheeses, where *Penicillium roqueforti* is able to colonize the formed eyes. However, production of these metabolites might be affected by phage infections. *Leuconostoc* phages cause failures during the fermentation of several foods, including wine, various types of cheese (blue cheese, Camembert, Cottage, Edam, Cream cheese), and other dairy products [27,48]. In comparison with lactococcal phages [15], there is scarce knowledge about thermal and chemical inactivation of *Leuconostoc* phages.

#### 2.2.1. Thermal Treatments

Bacteriophages attacking *Leuconostoc mesenteroides* and *Leuconostoc pseudomesenteroides* strains, which also contaminate raw milk during milk collection in dairy farms, may overcome the hurdle of milk pasteurization. In this sense, a study achieved on six *Leuconostoc* phages [40] demonstrated a high resistance in RSM, TMG buffer, and MRS (Biokar, Beauvais, France) broth when used as suspension media in pasteurization conditions at 63 and 72 °C. At 63 °C, the most sensitive phage (CyC) showed high viability (≥10^4^ PFU/mL) after 45 min of treatment. In turn, at 72 °C, this phage was inactivated completely after 5 min of treatment, whereas the most resistant phages were not completely inactivated even after 45 min at the same temperature. In this study, two high temperatures not frequently used in industry were tested: 80 °C and 90 °C. At 80 °C, the most resistant phages were completely inactivated only after 30 min of treatment in RSM; on the other hand, incubation at 90 °C for only 2 min was enough to achieve counts of <10 PFU/mL for all *Leuconostoc* phages tested in all suspension media. Moreover, Atamer et al. [49] evaluated the thermal resistance of 66 *Leuc. pseudomesenteroides* phages and 11 *Leuc. mesenteroides* phages in RSM, and demonstrated that approximately 98% of the phages studied survived after 1 min at 75 °C, and 70% of the phages were able to survive, at high numbers, at 80 °C for 1 min. In turn, when inactivation kinetics were performed for the phage with higher thermoresistance (*Leuconostoc* phage P793), they were still active after heating at 80 °C for 30 min in RSM. On the other hand, approximately 4% of all phages survived after 1 min at 85 °C [49]. According to previous studies, the low temperature long time pasteurization (LTLT) or the high temperature short time pasteurization (HTST) are not sufficient to guarantee the complete inactivation of *Leuconostoc* phages, allowing them to propagate and accumulate in dairy environments [40,49,50]. Considering the most resistant phages of both studies (CHA and P793), heat treatments at 85 °C for 70 s or 90 °C for 60 s would be required to completely inactivate all phages (Table 4).

As mentioned before, whey powder and whey components may be recycled and added to milk, thus improving texture and/or nutrient values. This may, however, cause new fermentation disturbances due to surviving phages in the whey-derived powders or components [23,51]. In this sense, Wagner et al. [26], showed the stability of *Leuconostoc* phages, isolated from whey powder, during long term storage, revealing high stability at room temperature for 4 years. At the same time, a viability study confirmed the premise that phages isolated from spray-dried whey powders may have higher heat resistance than phages isolated from dairy samples [27]. All the phages (32 phages) isolated frin whey powders were subjected to 1 min thermal treatment, obtaining 50% inactivation at 80 °C and complete inactivation at 85 °C. In turn, when the kinetic inactivation parameters (n, E_A_, k_ref_) of the *Leuconostoc* phage P973 (isolated from a whey powder sample) were analyzed and compared with the kinetic parameters and inactivation rates of the P793 phage (isolated from cheese), the greatest resistance of the isolated phages from the whey powder was reconfirmed [49]. As can be seen in these studies, there is a clear tendency for whey powder-derived phages to survive harsh thermal conditions in higher percentage proportions. Considering the data obtained with phage P973, its complete inactivation in milk would require a holding time of 20 min at 85 °C or 3 min at 90 °C. All these data agree with the suggestions of Wagner et al. [27], who pointed out that whey powders must be carefully considered as constituents for dairy fermentation processes as they represent a risk for the activity of starter cultures.

#### 2.2.2. Chemical Treatments

Pujato et al. [40] evaluated the chemical resistance of six *Leuconostoc* phages against different biocides (Table 2). In this study, ethanol was tested at three different concentrations, 50%, 75%, and 100% (v/v). Ethanol solution at 75% produced the highest phage inactivation, while the lower concentration (50%) did not produce, in general, significant effects. In addition, the inactivation efficiency of sodium hypochlorite on *Leuconostoc* phages was tested, and the effectiveness observed for concentrations allowed in the food industry was low or moderate. The most sensitive phages required 600 ppm during 30 min to show a complete inactivation, while concentrations between 1400 ppm and 1600 ppm of residual-free chlorine were needed to achieve complete inactivation of the most resistant phages.

Like most dairy phages, those infective for *Leuconostoc* were inactivated completely after 2 min with different sanitizers commonly used in the food industry, specifically peracetic acid (0.15% v/v), quaternary ammonium chloride (biocide A, 0.50% v/v), alkaline chloride foam (biocide C, 2.5% v/v), and ethoxylated nonylphenol and phosphoric acid (biocide E, 0.8% v/v) [35,40] (Table 2). Because of the extreme pHs values (pH values lower than 2 and greater than 12) of these sanitizers, their inactivation ability might be due not only to their components but also to the pH value itself [40].

### 2.3. Streptococcus thermophilus Bacteriophages

*St. thermophilus* is a component of thermophilic starter cultures, widely used in the manufacture of dairy products (yogurt and several types of cheeses) due to their favorable acidification and texturizing properties [60]. Thus, it is the second most important dairy species after *L. lactis* [61]. As a consequence of the extensive industrial use of this bacterial species in fermentative processes, phages able to infect *St. thermophilus* strains have been isolated and widely characterized.

In general, *St. thermophilus* phages evidence similar morphology, with isometric capsids and long and non-contractile tails. Thus, they belong to the family *Siphoviridae* (*Caudovirales* order). Nowadays, phages infective to *St. thermophilus* can be classified into four groups [7,61]. Based on their DNA packaging mechanism (presence or absence of cohesive genomic extremities) and profiles of structural proteins (two or three major structural proteins), phages are classified into *cos* or *pac* groups, respectively [62]. In this sense, phages belonging to the well-studied groups *cos* or *pac* have evidenced a relative homogeneity among themselves. It is worth mentioning, that most *St. thermophilus* phages isolated from industrial environments are members of the *cos* group. In addition, it was reported that *cos*-type phages are strictly virulent whereas both virulent and temperate phages can be found in the *pac* group [7]. The third group is represented by the 5093 phage, which evidenced both genomic and morphological differences in comparison to those reported for the *cos* or *pac*-type *St. thermophilus* phages [63]. Thus, genetic analysis of phage 5093 showed high homology to the prophages of non-dairy streptococci and limited similarity with other sequenced *St. thermophilus* phages. Moreover, a baseplate of phage 5093 containing fibers with globular ends was found. The fourth group called 987, is characterized by phages with shorter tails (in comparison to those found for other *St. thermophilus* phage groups) and with a baseplate of similar characteristics to those evidenced in the P335 species *L. lactis* phages. Also, a hybrid genome architecture exhibiting DNA sequences similar to both *St. thermophilus* and lactococcal P335 phages was reported [64].

#### 2.3.1. Thermal Treatments

To our knowledge, the first study about the thermal resistance of *St. thermophilus* phages was reported by Deane et al. [53] (Table 4). Resistance of a phage isolated from the whey of Swiss cheese was studied at 61.7 and 71.7 °C for 64 min, using skim milk as a suspension medium. Reductions higher than 99% after 30 min and 2 min were obtained at 61.7 and 71.7 °C, respectively.

Afterwards, Sozzi and Marret [44] studied the thermal inactivation of phage s265 (isolated from whey from an Emmental factory) at 55, 65, 72, and 90 °C for 60 min, and complete phage inactivation (initial titers of 10^9^ PFU/mL) was obtained through these treatments, 30 min at 65 °C, 10 min at 72 °C, and 20 s at 90 °C.

A detailed study was reported by Binetti and Reinheimer [30]. Authors studied the thermal resistance (63, 72 and 90 °C for 45 min) of five phages (10^6^ PFU/mL) isolated from Argentinian dairy products (cheese whey and yogurt) in three suspension media: ETS broth (tryptic soy broth added of 0.5% w/v yeast extract, 0.5% w/v lactose and 0.02% w/v L-cysteine), RSM, and a TMG buffer. Although the results were dependent on the phage studied, all virions showed resistance against pasteurization treatments commonly applied in the dairy industry (63 °C for 30 min and 72 °C for 15 s), similar to results found for the St. thermophilus phages described above [44,53]. Moreover, none of the five phages were completely inactivated at 63 °C after 45 min of treatment in all suspension media. Regarding the treatment at 72 °C, only two phages completely lost their infectivity within 30 min. Although an effect of the suspension medium on the sensitivity/resistance of phages could be observed, the results were not able to verify evident differences about the suspension media. Thermal inactivation curves (at 63 and 72 °C) of these phages are composed by two steps: a high decrease of phage infectivity at the early stages followed by a reduced rate of phage inactivation. This behavior could be attributed to the heterogeneity of the viruses (composed by phage particles with different thermal resistance) or due to the physical phenomenon of clumping. Finally, no phage was able to survive the treatment at 90 °C for 5 min in any suspension media assayed.

As previously mentioned, a Standard IDF microbiological methodology, which includes a treatment for 15 min at 90 °C (to discriminate between phage and non-phage inhibitors), is normally applied to dairy samples suspected of phage contamination. On the basis of the high thermal resistance evidenced for same dairy phages in the last years, Capra et al., [57] revised the effectiveness of the mentioned treatment in phage inactivation, studying dairy phages (specific for *L. lactis*, *St. thermophilus*, and lactobacilli) with high thermal resistance. Investigations included four *St. thermophilus* phages (031-D, CGL-3, CGL-4 and WPC1) in the final concentrations of 10^7^ PFU/mL, which were subjected to treatment at 90 °C for 45 min in skim milk. Phage 031-D was the most sensitive (among *St. thermophilus* phages), since it reached its complete inactivation within 10 min. On the other hand, the remaining phages were able to survive the treatment at 90 °C for 15 min (in the titers until 10^4^ PFU/mL), but they were completely inactivated within 40 min. Among the phages studied, *St. thermophilus* phages evidenced a higher thermal resistance than lactobacilli phages, even though it was lower than for *L. lactis* phages [57]. Since heating at 90 °C for 15 min would be not enough to inactivate all phages tested, the authors proposed to extend the treatment time to 45 min.

As mentioned previously, dairy phages have been detected in whey and/or their by-products, which are usually added to the manufacturing processes of dairy products. In this sense, some authors have reported the presence of phages infective to *St. thermophilus* in these type of samples with a subsequent characterization of the phages. Thus, Wagner et al. [26] analyzed samples of whey powders and whey powder formulations in relation to the presence of dairy bacteriophages. *St. thermophilus* phages were found in eight out of thirteen whey powders samples and in one out of ten whey powder formulations. In total, nine phages were isolated from samples, in titers ranging from 10 to 10^5^ PFU/g using *St. thermophilus* strains previously isolated from dairy samples and starter cultures. These nine phages had a similar morphology, with isometric heads and long non-contractile tails, being classified within *Shipoviridae* family. Moreover, all phages were the *cos*-type, as was reported for most of dairy phages infective to *St. thermophilus* [65]. In a subsequent work, these phages (10^5^–10^7^ PFU/mL) were evaluated for their thermal inactivation (1 min at 50–85 °C) in skim milk [27]. In this way, all phages were inactivated by heating at 85 °C and just one phage did not survive at 80 °C. To continue the investigation, one *St. thermophilus* phage was selected on the basis of heat stability, in order to determine the kinetic parameters of thermal inactivation (65–97.3 °C up to 90 min). Thus, the complete phage inactivation was only reached after 20 min (from initial concentration of 10^8^ PFU/mL) at 80 °C in milk, whereas a 10 min treatment was required to obtain the same behavior in water. As described above, a protective effect of milk against heat was also observed in this case [27]. Compared to the results obtained for other dairy phages (infective to *L. lactis* and *Leuconostoc*) in the same study [27], *St. thermophilus* phages seem to be the most sensitive to thermal treatments, although complete inactivation is not possible through a standard pasteurization process (72 °C for 15 s).

#### 2.3.2. Chemical Treatments

Regarding the biocide treatments (Table 2), Sozzi and Marret [44] studied the effect of sodium hypoclorite (0.05%, 0.01%, and 0.005%) on phage s265 inactivation. This phage remained infective after 30 min at 0.005% and 0.01%, whereas 5 min were enough to reach the complete inactivation of phage at 0.05% of sodium hypoclorite. Later, sodium hypochlorite solution (3.3–3.4% w/v available chlorine), a commercial disinfectant composed of peracetic acid (10% w/v), and hydrogen peroxide (30% w/v) were assayed on phage P53 [45]. Trials were done in the absence or presence of organic compounds (skim milk and an acidic whey solution, in a final concentration of 1% v/v) and for a contact time of 5, 15, 30 or 60 min. Results showed a protective effect of organic compounds on phage inactivation. In this sense, concentrations of 0.56% of sodium hypoclorite and 20 ppm of the commercial disinfectant were required to reach a phage infectivity reduction of 6 log orders within 15 min, in the absence of organic compounds. However, concentrations of 1.4% and 3.2% (in the presence of whey and milk, respectively) and 200 ppm (in presence of whey) and 50 ppm (in the presence of milk) of the commercial disinfectant were necessary to obtain the same inactivation (similar reduction and contact time) as the inactivation in the absence of organic compounds. Thus, according to the results obtained in this work, it would be suitable to test the efficiency of disinfectants in similar conditions to those in which they are used.

On the other hand, the effectiveness of sodium hypochlorite (100–200 ppm), ethanol (10–100% v/v), isopropanol (10–100% v/v), and peracetic acid (0.15% v/v) were investigated for five indigenous *St. thermophilus* phages by exposure for 45 min [30]. The most effective biocides in the inactivation of these phages were sodium hypochlorite (100 ppm) and peracetic acid (0.15%), since no phage particles were detected after 5 min of treatment. In addition, concentrations of 75% and/or 100% of ethanol reached a complete inactivation of three out of five phages within 15 and 45 min (depending on the phage). Isopropanol had a minor effect in phage inactivation.

In a more recent work, the effectiveness of five chemical products (with relevance in the food and dairy industry) in the inactivation of phage 2972 (isolated from yogurt sample) was evaluated [28]. These five sanitizers were previously chosen among a total of 23 chemical products based on their efficiency in the inactivation of phage P008 (infective to *L. lactis* IL1403 strain). Thus, phage 2972 was exposed to 0.2% of Oxi-B (peracetic acid, 5–10%; acetic acid, 7–13%; H_2_O, 15–40%), Oxi-D (0.13%), 200 ppm of QAC (benzalkonium chloride, 10–20%), 200 ppm of Anionic-B (phosphoric acid, 10–30%; octanoic acid, 1–5%; lactic acid, 1–5%; propylene glycol, 5–10%) and 200 ppm of Anionic-D (phosphoric acid, 15–40%; oleic acid, sulfonated, sodium salt, 7–13%). The composition of these sanitizers was previously described in the *L. lactis* section (Table 2). Exposure times were 2 and 15 min, and the experiments were carried out in the presence of 1% v/v of milk. The five sanitizers effectively inactivated phage 2972, with no detected virions (reductions of >4.6 log orders) in the presence of all biocides after 15 min of contact. The same behavior with 2 min of exposure for chemicals Oxi-D and Anionic-B was observed. On the other hand, for Oxi-B, QAC and Anionic-B, decreases of 4.6, 4.2, and 4.1 log orders in phage titers after 2 min of treatment were obtained, respectively. In addition, and considering that phages could remain in drains, the authors studied the effect of Bromochlorodimethylhydantoin (BCDMH), a product used commonly as a water disinfectant (100 ppm in the presence of 1% milk). After 2 min of exposure, the phage titers fell approximately 4 log orders, thus demonstrating the effectiveness of BCDMH on phage inactivation.

### 2.4. Lactobacillus Bacteriophages

As previously mentioned, information about phage infections in strains of the *Lactobacillus* genus (taking into account all species used in the manufacture of dairy products) is still relatively low compared to those affecting lactococci and *St. thermophilus* [4]. However, considering the industrial relevance of the starter cultures composed by lactobacilli strains, bacteriophages that infect strains of this genus are considered a major economic problem worldwide in food fermentation. These phages have been characterized mainly by their genomes and their interactions with sensitive bacterial strains, but data on the chemical and thermal resistance of phages of each species are still scarce.

#### 2.4.1. Thermal Inactivation

Some investigations about the thermal inactivation of phages infective to lactobacilli have been reported. These studies included phages specific for all the *Lactobacillus* species with industrial importance. Moreover, most of these phages have been isolated from samples of dairy industries that evidenced slow acidification. Studies mainly included thermal treatments usually applied to the milk destined for cheese or yogurt manufacture, that is 63, 72, and 90 °C (Table 4). Treatments were applied for several minutes to phage suspensions, with titers ranging from 10^6^ to 10^7^ PFU/mL. These studies demonstrated, as discussed for phages infective to other LAB genera, traditional pasteurization treatments (LTLT, 63 °C for 30 min and HTST, 72 °C for 15 s) would be not enough to reach complete inactivation of dairy phages, since most of them survive (in diverse levels) at these conditions. However, no phage particles were detected at 90 °C for 5 min, except for some phages infective to *Lactobacillus delbrueckii* [32,38] and *Lactobacillus casei*/*paracasei* [58,59], which keep their infectivity until 30 min at this temperature (depending on the phage). On the other hand, phages infecting lactobacilli showed diverse responses (phage-dependent behavior) when they were subjected to treatments at 63 and 72 °C for 45–60 min. Moreover, some investigations included trials performed in diverse suspension media in order to evaluate the potential protective effect of some components of the media on phage particle inactivation [32,33,35,36,43,66]. Thus, media used in dairy processes (e.g., milk and a commercial medium to propagate probiotic bacteria), as well as laboratory media, such as an MRS broth (Biokar, Beauvais, France) and a TMG buffer, were studied. In this way, a higher thermal resistance in some media (mainly skim milk) during the application of thermal treatments for phages infective to *Lb. delbrueckii* [32,35] and *Lb. casei*/*paracasei* [33,36,43] was evidenced.

Specifically, for phages infective to *Lactobacillus helveticus*, Sozzi and Maret [44] reported that treatments of 1 h at 72 °C and 20 s at 90 °C were required to inactivate phage l112. Afterwards, Quiberoni et al. [31] carried out investigations about the thermal inactivation of four phages: hv, ATCC 15807-B1, CNRZ 832-B1, and CNRZ 024. Results demonstrated a behavior dependent on the phage, since two phages were still infective after 45 min at 63 and 72 °C (Table 4). As it was described for some *St. thermophilus* phages [30], the thermal inactivation kinetics of *Lb. helveticus* are composed by two or three linear components, i.e., a rapidly inactivated phage component followed by a slower one, due to the inherent heterogeneity of the virus [31].

Regarding the *Lb. delbrueckii* phages, Quiberoni et al. [32] studied the thermal inactivation of one collection phage (LL-H) and three phages isolated from yogurt plants: phages YAM and Ib_3_ (infective to *Lb. delbrueckii* subsp. *lactis*) and phage BYM (infective to *Lb. delbrueckii* subsp. *bulgaricus*). The results demonstrated a phage-dependent behavior, with the collection phage being the most heat-sensitive. Phages BYM and Ib_3_ were highly resistant at 63 °C but were completely inactivated after 30 and 45 min at 72 °C, respectively. For phages YAB and LL-H, no infective particles were observed at 72 °C, after 15 min (phage YAB) or 5 min (phage LL-H) of treatment. For the four phages, a clear protective effect of skim milk was observed at the two temperatures, reaching a lower resistance in the MRS broth (Biokar, Beauvais, France) (used as suspension medium). Thus, in the presence of skim milk, it was not possible to obtain a complete inactivation of phages (whereas in MRS it was reached) or longer times were required for the complete loss of phage infectivity, depending on the phage/temperature studied. It should be noted that phage Ib_3_ evidenced a marked heat resistance, since a treatment of 15 min at 90 °C was necessary to reach complete inactivation when skim milk was used as a suspension medium. In the case of the MRS broth or TMG buffer, 5 min at 90 °C was enough. On the other hand, Ebrecht et al. [35] studied the resistance of two temperate phages (Cb1/204 and Cb1/342, released from a *Lb. delbrueckii subsp. lactis* commercial strain used in dairy industries) against traditional thermal treatments (63, 72 and 90 °C), in two suspension media (skim milk and MRS broth). In this sense, high-titer suspensions of the temperate phages were inactivated at 63 °C after 20 min in the MRS broth (Biokar, Beauvais, France) but phage particles (10^2^ PFU/mL) were detected after 30 min in the presence of skim milk, evidencing a protective effect of the milk. No phage particles were detected for both phages with treatments at 72 °C for 5 min, except for phage Cb1/204, which required 20 min for complete inactivation in skim milk. On the other hand, treatments at 82 and 90 °C for 2 min were enough to completely inactivate both phages. The temperate phages, which have prolate heads, seem more heat-sensitive in comparison to phages YAB, Ib_3_, and BYM (all with isometric heads) studied by Quiberoni et al. [32]. In a similar way, a high heat-sensitivity was reported for the temperate phage lb539 [52]. In this way, a reduction of 90%, 99.9% and 99.99% of phage particles at 50, 60, and 70 °C, respectively, after 60 min of treatment was reported. On the other hand, Wang et al. [54] studied the thermal inactivation of a virulent phage phiLdb (isolated from Chinese yogurt sample) in MRS broth (Biokar, Beauvais, France). This phage evidenced a high heat-resistance in a temperature range from 60 to 90 °C. Similar to phage Ib_3_ [32], the phage phiLdb showed high resistance at 90 °C, requiring 40 min for its complete inactivation [54] (Table 4).

In the case of *Lb. casei*/*paracasei* phages, Capra et al. [33] found that two collection phages (J-1 and PL-1) were inactivated by heating at 63 °C (within 45 min) and 72 °C (within 15 min) for most conditions studied. Similarly, two temperate phages (i*Lp*84 and i*Lp*1308) isolated from the induction of *Lb. paracasei* strains 84 and CNRZ1308, respectively, were studied by Mercanti et al. [43]. Phage i*Lp*84 evidenced a lower heat resistance than phage i*Lp*1308, since a complete inactivation was achieved after 30 min at 72 °C and 45 min at 63 °C. On the contrary, phage i*Lp*1308 was completely inactivated after 45 min (at 63 and 72 °C) in MRS broth (Biokar, Beauvais, France), whereas reductions of 1 (63 °C) and 2 (72 °C) log orders were obtained in skim milk. Thus, a protective effect of media was evidenced for this phage [43]. On the other hand, a high thermal resistance was demonstrated by phage MLC-A, isolated from a probiotic dairy product. In this sense, this phage was able to survive treatments at 63 and 72 °C for 45 min [36]. Likewise, for phage i*Lp*1308, skim milk exerted a protective effect in the MLC-A inactivation [36]. Moreover, for some phages infecting this bacterial species, an extreme heat-resistance was reported. In this sense, some phages were able to survive treatments at 90 °C for several min. Specifically, phages C_L_1 and C_L_2 (isolated from noninfected lysed-cultures of a commercial *Lb. paracasei* strain) required 15 or 30 min to reach their complete inactivation at 90 °C, depending on the suspension media/phage studied [58]. Furthermore, some phage particles were detected after 30 min at 90 °C when phage C_L_2 was tested in skim milk. A similar behavior was reported by Capra et al. [59], by testing eight phages isolated from an industrial plant that manufactures a probiotic dairy product. Phages were inactivated by heating at 90 °C after 15 or 30 min of treatment (depending on the suspension media studied). Moreover, three phages were able to survive the heating at 90 °C for 30 min, mainly when skim milk or an EM medium a (commercial medium used to propagate probiotic strain) were evaluated, demonstrating a protective effect once more. On the other hand, a lower thermal resistance was observed for a non-dairy phage (Lcb), isolated from Chinese sauerkraut when its thermal resistance was studied in MRS broth (Biokar, Beauvais, France). In this way, treatments at 70 °C for 30 min or at 80 °C for 10 min were enough to inactivate the entire phage particle population [38] (Table 4). As mentioned for *St. thermophilus* phages, those infective of *Lb. casei*/*paracasei* were studied for their resistance to the thermal treatment included in a Standard IDF microbiological methodology used for phage detection in dairy samples [57]. Thus, phages J-1, C_L_2, and MLC-A3R were exposed at 90 °C for 45 min in skim milk. Except for phage J-1 (which was inactivated after 10 min of treatment), *Lb. casei*/*paracasei* phages were able to survive the treatment (90 °C for 15 min) suggested by the IDF for phage detection. Moreover, phages C_L_2 and MLC-A3R were counted (in low titers) after 30 min, whereas a complete inactivation was reached after 40 min. Based on these results, it would be valuable to revise the conditions (temperature and time) of the thermal treatment included in the IDF methodology in order to achieve complete inactivation of dairy phages.

Regarding the *Lactobacillus plantarum* phages, Briggiler Marcó et al. [34] reported a strong resistance of four phages (ATCC 8014-B1, ATCC 8014-B2, FAGK1, and FAGK2) at 63 °C for 45 min whereas significant phages particles reductions (4 and 5.5 log reductions after 45 min of treatment), but without reaching a complete inactivation, were observed at 72 °C. In recent studies, two phages (P1 and P2) isolated from an abnormal broth fermented with *Lb. plantarum* IMAU10120, showed a lower heat resistance since they were completely inactivated at 63 and 70 °C within 40–50 min and 10–20 min, respectively [37,39]. In both cases, a slightly higher resistance (protective effect) in media usually used in the dairy industry (milk skim and EM medium) was evidenced in comparison to the results obtained in laboratory media: the TMG buffer and MRS broth (Biokar, Beauvais, France) [34,37,39]. On the other hand, studies the thermal resistance of non-dairy phages infective to *Lb. plantarum* have also been reported [55,56]. Thus, phage JL-1 (isolated from cucumber fermentation) was completely inactivated in water by heating at 80 °C (20 s) or 90–100 °C (15 s) [56]. A lower thermal resistance was evidenced for phage fri (isolated from a meat starter culture). In this case, only 30% of phage particles resisted a treatment for 5 min at 60 °C, as no phage particles were detected after 10 min at 60 °C or 5 min at 70 °C [55] (Table 4).

#### 2.4.2. Chemical Inactivation

Phages infective to the lactobacilli species commonly used in food processes have been also investigated for their resistance against sanitizers (Table 2). The investigated biocides included those usually applied in dairy to sanitize the environment, the equipment, and laboratories. Among these traditional biocides, ethanol (10–100% v/v), isopropanol (10–100% v/v), sodium hypochlorite (100–1400 ppm), and peracetic acid (0.15% v/v) were investigated for inactivation of phage suspensions (10^6^–10^9^ PFU/mL). In this way, peracetic acid (0.15% v/v) was the most effective biocide for all investigated phages since it completely inactivated high titer phage suspensions in a short time (5 min) [31,32,33,34,35,36,43], with the exception of phages P1 and P2 (specific for *Lb. plantarum* strains). For these phages, large phage particle numbers were found after 60 min, even at concentrations of 0.45% for this biocide [37,39]. On the contrary, alcohols in general showed low efficiency for phage inactivation. In this sense, concentrations of 10% had no effect on phage particle inactivation, whereas for higher concentrations, the results obtained were diverse. In the case of sodium hypochlorite, its behavior was dependent on the phage, but, in general, complete inactivation was reached with concentrations higher than those allowed in food industries (200 ppm). Thus, the use of this biocide at concentrations assuring phage inactivation in an industrial environment would not be possible. Besides otraditional biocides, the inactivation efficiency of new commercial sanitizers was studied on phages infective to *Lb. delbrueckii*, *Lb. casei*, and *Lb. plantarum* strains [28,35,43].

Regarding phages specific for *Lb. helveticus*, the inactivation of phage l112 was studied in presence of sodium hypoclorite (0.05%, 0.01%, and 0.005%) for contact times up to 30 min [44]. In this sense, concentrations of 0.05% produced the complete infectivity loss of the phage within 5 min, whereas phage suspension was partially reduced in the presence of 0.01% and 0.005% sodium hypoclorite after 30 min. Additionally, Quiberoni et al. [31] found a high effectiveness of hypochlorite sodium in concentrations of 100 ppm. Thus, the four studied phages completely lost their infectivity within 5 min (phages hv and CNRZ 0241) and 10 min (phages ATCC 15807-B1 and CNRZ 832-B1) of contact. Regarding ethanol, a concentration of 75% (v/v) was the most effective, reaching complete inactivation within 5–15 min of treatment. On the other hand, concentrations of 50% produced the same effect a 100%. In this sense, no phage particles (<10 PFU/mL) were detected after 5–15 min of treatment, except for phage hv, for which a partial inactivation (3–4 log reductions) was reached after 45 min. A phage-dependent behavior was observed when isopropanol was assayed. Two phages (CNRZ 832-B1 and CNRZ 0241) were completely inactivated with 50% and 100% isopropanol, respectively, within 5 min, whereas the complete inactivation of phage ATCC 15807-B1 was reached in the presence of 100% isopropanol after 45 min. On the other hand, a partial reduction was observed for phage hv in the presence of 50% (2 log reductions) and 100% (4 log reductions) isopropanol. On the other hand, 10% isopropanol had little effect on phage viability (0.2–1.5 log reductions) in all cases.

In the case of *Lb. delbrueckii* phages, Quiberoni et al. [32] found that alcohols (ethanol and isopropanol) had little influence on the viricidal activity of the four phages (YAB, BYM, Ib_3_ and LL-H). In this sense, 100% ethanol produced the fastest inactivation of phage particles. Little or no effects were observed when 10% and 50% ethanol were assayed. Similar results were obtained in the presence of isopropanol. Results obtained in the presence of sodium hypochlorite were dependent on the phage. In this sense, phage Ib_3_ showed a very high resistance, since concentrations of 1200–1400 ppm of residual-free chlorine were needed to achieve its complete destruction after 45 or 15 min, respectively. On the other hand, for the remaining three phages, 300–400 ppm of sodium hypochlorite produced total inactivation within 5–45 min of contact (depending on the phage). Afterwards, Ebrecht et al. [35] studied the chemical inactivation of two temperate phages (Cb1/204 and Cb1/342) in the presence of traditional and new commercial sanitizers. Ethanol concentrations between 10% and 75% produced only 1 log reduction in the titers of both phages. However, 100% ethanol achieved complete inactivation for phage Cb1/204 after 45 min, whereas the phage suspension of about 10^4^ PFU/mL remained viable after 45 min for phage Cb1/342. Moreover, isopropanol was less effective than ethanol. Regarding sodium hypochlorite, phage Cb1/342 evidenced higher resistance than phage Cb1/204 since 300 ppm and 200 ppm, respectively, were necessary for total inactivation within 2 min of exposition. In addition, complete inactivation was reached in the presence of 100 and 200 ppm of sodium hypochlorite within 30 min for both phages. The new commercial sanitizers tested were biocides A (quaternary ammonium chloride, 0.5–3.0% v/v for until 20 min), B (hydrogen peroxide, peracetic acid and peroctanoic acid, 0.13%, 0.26%, v/v for until 20 min), C (alkaline chloride foam, 2.5%, v/v for until 45 min), D (pure until 45 min), E (ethoxylated nonylphenol and phosphoric acid, 0.8%, v/v for until 20 min), and the study of efficiency of these biocides also included phages BYM, YAB, and Ib_3_ (previously mentioned in [45]). Thus, biocide A in a concentration of 0.5% produced complete inactivation of phages BYM and YAB after 20 min, whereas the three remaining phages remained in a concentration of about 10^5^–10^6^ PFU/mL after the same time. However, for all phages, no phage particles were detected after 2 min in the presence of this biocide in concentrations of 1%. Similar results were found for biocides C and E, since no phage particles were detected after 2 min of treatment, mainly due to the extreme pH of these biocides (pH values of 12.4 for biocide C and of 2.0 for biocide E). Biocide B (0.13%) rapidly inactivated all phages, with the exception of phage Ib_3_ which required 10 min for its complete inactivation with concentrations of 0.26%. On the other hand, biocide D showed low efficiency to inactivate phage particles, since reductions lower than 3 log orders after 45 min were obtained in all cases.

The first investigations about the chemical inactivation of phages infective to *Lb. casei* were carried out in about 1970. In this sense, Watanabe et al. [41] studied the influence of ethanol (30%) and sodium hypochlorite (0.1–1 mg/mL) for 60 min on infectivity of the collection phage PL-1. Thus, a viricidal effect was reported in the presence of ethanol and 1 mg/mL of residual-free chlorine, whereas a 1 log reduction was obtained when 0.1 mg/mL of sodium hypochlorite was assayed. Afterwards, Lee et al. [42] found that the same phage required ethanol concentrations higher than 30% (for 20 h) to affect the plaque formation. Additionally, Capra et al. [33] studied the collection phages PL-1 and J-1. The authors demonstrated that high concentrations of sodium hypochlorite (800 ppm) were required to reach a total inactivation of both phages (within 5 min). On the other hand, the remaining concentrations (400, 600, and 700 ppm) caused a partial phage inactivation within 45 min. Alcohols (ethanol and isopropanol) were not very efficient as virucidal agents for the two phages. A similar behavior was observed for phage MLC-A, isolated from a probiotic dairy product [36]. The viability of this phage was not altered by the presence of isopropanol (10–100%) and low ethanol concentrations (10% and 50%). However, a partial reduction (3.2 log orders) was observed with 75% ethanol after 45 min, making this treatment the most effective among alcohols. Phage MLC-A required 700 ppm of sodium hypochlorite for 30 min to reach a complete inactivation. Mercanti et al. [43] studied the effect of classic (ethanol and sodium hypochlorite) and the new commercial sanitizers mentioned above (A, B, C, D, E) on the inactivation of two temperate phages (i*Lp*84 and i*Lp*1308). Phages evidenced a dissimilar behavior in the presence of sodium hypochlorite, although both were completely inactivated. Thus, concentrations of 400 ppm for this biocide for 5 min were necessary to inactivate phage i*Lp*1308, whereas 800 ppm and 30 min were required for i*Lp*84. However, the inactivation of both phages in the presence of ethanol was similar. Thus, 75% ethanol was the most effective for phage inactivation, although a complete inactivation of phages was not reached. In the case of the new commercial sanitizers, biocide B (0.13% and 0.26% v/v for 20 min) and D (pure for 120 min) were not suitable for the elimination of phages i*Lp*84 and i*Lp*1308. On theother hand, biocide A was highly efficient for phage inactivation, since a concentration of 0.5% v/v produced a complete destruction of phages within 5 min of treatment. Moreover, concentrations of 0.25% v/v of biocide A quickly inactivated phage i*Lp*84 (15 min for total inactivation), but titers of phage i*Lp*1308 only decreased 1 log order after 45 min of incubation. On the other hand, high efficiency for the inactivation of phages i*Lp*84 and i*Lp*1308 was demonstrated by biocides C (0.25% v/v) and E (0.8% v/v), which achieved a complete inactivation within 5 min of incubation. Recently, Zhang et al. [38] studied diverse isopropanol and ethanol concentrations on inactivation of phage Lcb, isolated from Chinese sauerkraut. No phage particles were detected after 10 min (100% of both alcohols) and 20 or 30 min in presence of 75% ethanol and isopropanol, respectively.

For *Lb. plantarum* phages, the four phages studied (ATCC 8014-B1, ATCC 8014-B2, FAGK1 and FAGK2) [34], evidenced a similar behavior in presence of diverse biocides. Infectivity of *Lb. plantarum* phages was scarcely affected by isopropanol (10–100% v/v) and ethanol (10% v/v). A better performance was obtained in presence of ethanol (50–100%) but without reaching a complete inactivation of phages. On the contrary, a complete loss of viability for the four phages in presence of sodium hypochlorite was obtained. Thus, 800 pm produced a complete phage inactivation within 15 and 30 min of contact, depending on the phage. However, 200 and 400 ppm of this sanitizer produced a partial destruction of the phages. In addition, phage ATCC 8014-B1 was studied in a subsequent work about its sensitivity against five commercial sanitizers (previously selected based on their efficiency in phage P008 inactivation) [28]. Phage ATCC 8014-B1 was exposed to the sanitizers for 2–15 min (previously described in the lactococci section): Oxi-B (0.2%), Oxi-D (0.13%), QAC (200 ppm), Anionic-B (200 ppm) and Anionic-D (200 ppm), in the presence of 1% v/v of milk. Except for Anionic-B, all sanitizers produced reductions >4 log orders of phage ATCC 8014-B1 within 2 min of contact time, demonstrating them to be effective in phage inactivation. On the contrary, a 3 log reduction after 15 min of treatment was obtained in presence of Anionic-B. In a similar way, phages P1 and P2 (isolated from a slow fermentation containing *Lb. plantarum* IMAU10120) were studied for their resistance to chemical products [37,39]. Here, phage P1 showed higher resistance to sodium hypochlorite, since 800 ppm were needed for its complete inactivation within 60 min [37]. However, the inactivation of phage P2 was reached with concentrations of 800 and 400 ppm of residual chlorine free within 30 and 50 min, respectively [39]. Alcohols (ethanol and isopropanol) were not effective for total inactivation of both phages, since phage populations higher than 10^2^ PFU/mL remained viable after 60 min in the presence of 100% of both alcohols. An unexpected behavior was obtained for phages P1 and P2 in the presence of peracetic acid, which has been widely documented as efficient for the total inactivation of dairy phages in a short time (lower than 5 min). However, in the case of phages P1 and P2, the use of peracetic acid at 0.15% had little effect on the phage populations and reductions of 4 (phage P1) and 1.5 (phage P2) log orders after 60 min were obtained with concentrations of 0.45% of this biocide [37,39].

## 3. Concluding Remarks

The existing studies demonstrate that, in general, LAB bacteriophages are able to survive the thermal treatments usually applied in dairy for the sanitization of raw milk or those involved in technological processes, such as spray drying. In recent years, phages with increased and, in some cases, extraordinary thermal resistance were isolated, and this fact led to the need to revise the methodology applied to detect phages in industrial samples. Even though thermal treatments are very valuable tools in phage control, the temperature/time selection will depend of the desired characteristics of the final product and the technologies involved in each manufacture process. Critical thermal treatments required for the complete inactivation of highly thermo resistant phages are not practically applicable in dairy processes, as this would result in a pronounced deterioration of the final product.

Regarding the effectiveness of sanitizers on LAB phage viability, the available information reveals substantial variations depending on the chemical nature. On the other hand, phages with the most resistance against biocide activity show a better survival against a wide spectrum of sanitizing compounds. Consequently, a careful and regular examination of available biocides against emerging new phages is recommended by specialists, together with the implementation of a rotation system of compounds used with the aim of increasing efficiency. Also, and as usual, several points must be considered when selecting sanitizers: the antimicrobial activity, ease of application, low cost, lack of negative impact on the final product, and degradation into harmless final compounds.

As is known, all available methodologies used in dairy plants are not completely efficient when they are applied individually, and a combination of techniques is essential to obtain adequate phage control. On the other hand, other practical approaches could be applied when possible; these approaches could include adapted factory design, adequate ventilation, use of strains with improved resistance phage, programs of culture rotation, or modifications in the processes.

## Figures and Tables

**Table 1:** Thermal treatments (temperature, time, suspension medium) studied for inactivation of lactococcal phages.

Temperature	Number of Studied Phages (Phage Group if Available) (Treatment Time, Suspension Medium if Available)
50–55 °C	5 phages (c2 group) (30 min, whole fat milk) [11]
	7 phages (936 group) (30 min, whole fat milk) [11]
	5 phages (P335 group) (30 min, whole fat milk) [11]
	1 phage (c2 group) (120 min, M17 broth) [20]
	1 phage (936 group) (120 min, M17 broth) [20]
	31 phages (936 group) (1 min, UHT-skim milk) [27]
	1 phage (P335 group) (1 min, UHT-skim milk) [27]
60–65 °C	4 phages (45 min, TMG buffer, M17 broth, RSM) [18]
	5 phages (c2 group) (30 min, whole fat milk) [11]
	7 phages (936 group) (30 min, whole fat milk) [11]
	5 phages (P335 group) (30 min, whole fat milk) [11]
	1 phage (c2 group) (120 min, M17 broth) [20]
	1 phage (936 group) (120 min, M17 broth) [20]
	9 phages (c2 group) (5 min, skim milk) [21]
	31 phages (936 group) (1 min, UHT-skim milk [27]
	1 phage (P335 group) (1 min, UHT-skim milk [27]
	1 phage (936 group) (90 min, water, skim milk) [27]
	2 phages (936 group) (16.5–42 s, raw milk) [16]
	1 phage (c2 group) (16.5–42 s, raw milk) [16]
70–75 °C	4 phages (45 min, TMG buffer, M17 broth, RSM) [18]
	10 phages (15 min, M17 broth) [19]
	5 phages (c2 group) (30 min, whole fat milk) [11]
	7 phages (936 group) (30 min, whole fat milk) [11]
	5 phages (P335) group) (30 min, whole fat milk) [11]
	1 phage (c2 group) (120 min, M17 broth) [20]
	1 phage (936 group) (120 min, M17 broth) [20]
	9 phages (c2 group) (5 min, skim milk) [21]
	2 phages (936 group) (5 min, UHT-skim milk, water, Ca-LM17) [15]
	1 phage (936 group) (60 min, whey, whey protein concentrate, whey cream) [24]
	31 phages (936 group) (1 min, UHT-skim milk) [27]
	1 phage (P335 group) (1 min, UHT-skim milk) [27]
	1 phage (936 group) (90 min, water, skim milk) [27]
	2 phages (936 group) (16.5–42 s, raw milk) [16]
	1 phage (c2 group) (16.5–42 s, raw milk) [16]
80–85 °C	5 phages (c2 group) (30 min, whole fat milk) [11]
	7 phages (936 group) (30 min, whole fat milk) [11]
	5 phages (P335 group) (30 min, whole fat milk) [11]
	9 phages (c2 group) (5 min, skim milk) [21]
	11 phages (936 group) (30 min, RSM) [22]
	42 phages (936 group) (5 min, UHT-skim milk, water) [15]
	11 phages (c2 group) (5 min, UHT-skim milk, water) [15]
	3 phages (P335 group) (5 min, UHT-skim milk, water) [15]
	1 phage (936 group) (60 min, whey, whey protein concentrate, whey cream) [24]
	31 phages (936 group) (1 min, UHT-skim milk) [27]
	1 phage (P335 group) (1 min, UHT-skim milk) [27]
	1 phage (936 group) (90 min, water, skim milk) [27]
	2 phages (936 group) (16.5–42 s, raw milk) [16]
	1 phage (c2 group) (16.5–42 s, raw milk) [16]
90–100 °C	4 phages (45 min, TMG buffer, M17 broth, RSM) [18]
	10 phages (5 min, M17 broth) [19]
	4 phages (936 group) (5 min, UHT-skim milk, water) [15]
	1 phage (936 group) (60 min, whey, whey protein concentrate, whey protein) [24]
	1 phage (936 group) (90 min, water, skim milk) [27]
	2 phages (936 group) (16.5–42 s, raw milk) [16]
	1 phage (c2 group) (16.5–42 s, raw milk) [16]

**Table 2:** Biocides studied for inactivation of dairy phages.

Biocide	Number of Studied Phages (Phage Lactococcal Group if Available) (Biocide Concentration)						
	*Lactococcus lactis*	*Streptococcus thermophilus*	*Leuconostoc mesenteroides (Lm)*/*pseudomesenteroides (Lp)*	*Lactobacillus helveticus*	*Lactobacillus delbrueckii*	*Lactobacillus casei*/*paracasei*	*Lactobacillus plantarum*
Isopropanol	4 phages (10%, 50%, 100%) [18]	5 phages (10%, 50%, 100%) [30]		4 phages 10%, 50%, 100%) [31]	4 phages (10%, 50%, 100%) [32]	2 phages (10%, 50%, 100%) [33]	4 phages (10%, 50%, 100%) [34]
	10 phages (10%, 50%, 100%) [19]				2 phages (10%, 50%, 100%) [35]	1 phage (10%, 50%, 100%) [36]	1 phage (10%, 30%, 50%, 100%) [37]
	1 phage (936 group) (70%, 80%) [28]					1 phage (10%, 25%, 35%, 50%, 75%, 100%) [38]	1 phage (10%, 30%, 50%, 100%) [39]
	36 phages (936 group) (40%, 80%) [5]						
Ethanol	4 phages (10%, 25%, 50%, 100%) [18]	5 phages (10%, 50%, 75%, 100%) [30]	5 phages *Lm* (50%, 75%, 100%) [40]	4 phages (10%, 50%, 75%, 100%) [31]	4 phages (10%, 50%, 75%, 100%) [32]	1 phage (30%) [41]	4 phages (10%, 50%, 75%, 100%) [34]
	10 phages (10%, 50%, 75%, 100%) [19]		1 phage *Lp* (50%, 75%, 100%) [40]		2 phages (10%, 50%, 75%, 100%) [35]	1 phage (5–20%) [42]	1 phage (10%, 20%, 30%, 50%, 75%, 100%) [37]
	1 phage (936 group) (70%, 80%) [28]					2 phages (10%, 50%, 75%, 100%) [33]	1 phage (10%, 20%, 30%, 50%, 75%, 100%) [39]
	36 phages (936 group) (40%, 80%) [5]					1 phage (10%, 50%, 75%, 100%) [36]	
						2 phages (10%, 50%, 75%, 100%) [43]	
						1 phage (10%, 25%, 35%, 50%, 75%, 100%) [38]	
Sodium hypochlorite	4 phages (100, 200, 300 ppm) [18]	1 phage (0.005, 0.01, 0.05%) [44]	5 phages *Lm* (200–1600 ppm) [40]	1 phage (0.005, 0.01, 0.05%) [44]	4 phages (100–1400 ppm) [32]	1 phage (0.1–1%) [41]	4 phages (200, 400, 800 ppm) [34]
	11 phages (936 group) (200–800 ppm [22]	1 phage (0.24–3.2%) [45]	1 phage *Lp* (200–1600 ppm) [40]	4 phages (100, 400 ppm) [31]	2 phages (100, 200, 300 ppm) [35]	2 phages (400–800 ppm) [33]	1 phage (100, 200, 400, 800 ppm) [37]
	1 phage (936 group) (200, 500 ppm) [28]	5 phages (100, 200 ppm) [30]				1 phage (200, 400, 600 ppm) [36]	1 phage (100, 200, 400, 800 ppm) [39]
						2 phages (100, 400, 800 ppm) [43]	
Peracetic acid	4 phages (0.15%) [18]	5 phages (0.15%) [30]	5 phages *Lm* (0.15%) [40]	4 phages (0.15%) [31]	4 phages (0.15%) [32]	2 phages (0.15%) [33]	4 phages (0.15%) [34]
	11 phages (936 group) (0.015%) [22]		1 phage *Lp* (0.15%) [40]		2 phages (0.15%) [35]	1 phage (0.15%) [36]	1 phage (0.15%, 0.25%, 0.45%) [37]
						2 phages (0.15%) [43]	1 phage (0.15%, 0.25%, 0.45%) [39]
Biocide A: Quaternary ammonium chloride			5 phages *Lm* (0.25%, 0.5%) [40]		5 phages (0.5%, 1.0%, 1.5%, 2.0%, 2.5%, 3.0%) [35]	2 phages (0.25%, 0.5%, 1%, 2%) [43]	
			1 phage *Lp* (0.25%, 0.5%) [40]				
Biocide B: Hydrogen peroxide, peracetic acid and peroctanoic acid					5 phages (0.13%, 0.26%) [35]	2 phages (0.13%, 0.26%) [43]	
Biocide C: Alkaline chloride foam			5 phages *Lm* (2.5%) [40]		5 phages (2.5%) [35]	2 phages (2.5%) [43]	
			1 phage *Lp* (2.5%) [40]				
Biocide D: p-toluensulfonchloroamide, sodium salt					5 phages (pure) [35]	2 phages (pure) [43]	
Biocide E: Ethoxylated nonylphenoland phosphoric acid			5 phages *Lm* (0.8%) [40]		5 phages (0.8%) [35]	2 phages (0.8%) [43]	
			1 phage *Lp* (0.8%) [40]				
Biocide F: N-(3-aminopropyl)-N-dodecylpropane-1, 3-diamine			5 phages *Lm* (2%) [40]				
			1 phage *Lp* (2%) [40]				
Oxi B: Peracetic acid 5–10%; acetic acid 7–13%; H_2_O 15–40%	1 phage (936 group) (0.2%, 0.35%) [28]	1 phage (0.2%) [28]					1 phage (0.2%) [28]
	3 phages (936 group) (0.2%) [28]						
	1 phage (c2 group) (0.2%) [28]						
	2 phages (0.2%) [28]						
Oxi-D: Peracetic acid 3–7%; acetic acid 15–40%; H_2_O_2_ 5–10%; L-octanesulfonic acid, sodium salt 3–7%; octanoic acid 1–5%	1 phage (936 group) (0.13%, 0.25%) [28]	1 phage (0.13%) [28]					1 phage (0.13%) [28]
	3 phages (936 group) (0.2%) [28]						
	1 phage (c2 group) (0.2%) [28]						
	2 phages (0.2%) [28]						
QAC: Benzalkonium chloride 10–20%	1 phage (936 group) (200, 500 ppm) [27]	1 phage (200 ppm) [28]					1 phage (200 ppm) [28]
	3 phages (936 group) (0.2%) [28]						
	1 phage (c2 group) (0.2%) [28]						
	2 phages (0.2%) [28]						
Anionic-B: Phosphoric acid 10–30%; octanoic acid 1–5%; lactic acid 1–5%; propylene glycol 5–10%	1 phage (936 group) (200, 500 ppm) [28]	1 phage (200 ppm) [28]					1 phage (200 ppm) [28]
	3 phages (936 group) (0.2%) [28]						
	1 phage (c2 group) (0.2%) [28]						
	2 phages (0.2%) [28]						
Anionic-D: Phosphoric acid 15–40%; oleic acid, sulfonated, sodium salt 7–13%	1 phage (936 group) (200, 500 ppm) [28]	1 phage (200 ppm) [28]					1 phage (200 ppm) [28]
	3 phages (936 group) (0.2%) [28]						
	1 phage (c2 group) (0.2%) [28]						
	2 phages (0.2%) [28]						
Cl_2_-triazinetrione: Dichloro-S-triazinetrione, sodium salt, solid	1 phage (936 group) (200 ppm) [28]						
BCDMH: Bromochlorodimethylhydantoin 60–100%	2 phages (936 group) (100 ppm) [28]	1 phage (100 ppm) [28]					
Commercial disinfectant (peracetic acid, 10% w/v—hydrogen peroxide, 30% w/v)		1 phage (10–200 ppm) [45]					

**Table 3:** Biocides studied for inactivation of lactococcal phages.

Biocide	Number of Studied Phages (Lactococcal Phage Group) (Biocide Concentrations)
**Chlorinated agents:** Chlorine dioxide	1 phage (936 group) (20, 50 ppm) [28]
**Peroxide and peroxyacid mixtures** (Peracetic acid%; acetic acid%, H_2_O_2_%)	
Oxi-A: 5–10%; 10–20%; 10–20%	1 phage (936 group) (0.2, 0.5%) [28]
Oxi-C: 15–17%; 33–38%; 9–11%	1 phage (936 group) (0.5, 1.4%) [28]
Na^2+^ percarbonate: sodium percarbonate 30–60%, solid	1 phage (936 group) (200, 500 ppm) [28]
**Quaternary ammonium compounds** (Benzalkonium chloride%; others)	
QAC+EtOH: 7–13%; ethanol 1–5%	1 phage (936 group) (200, 500 ppm) [28]
**Anionic acids** (Phosphoric acid%; others)	
Anionic-A: 10–30%; oleic acid, sulfonated sodium salt 1–5%; propylene glycol 3–7%	1 phage (936 group) (200, 500 ppm) [28]
Anionic-C: 10–30%; dodecyl benzene sulfonic acid 1–5%	1 phage (936 group) (200, 500 ppm) [28]
Anionic-E: 10–30%; 2-hydroxypropanoic acid 1–5%; sodium alkylnaphtalene sulfonate 10–30%; octanoic acid 3–7%; decanoic acid 0.5–1.5%	1 phage (936 group) (2000, 5000 ppm) [28]
**Iodine-based acids (Iodine; others)**	
Iodine-A: 1–5%; nitric acid 20–30%	1 phage (936 group) (25, 50 ppm) [28]
Iodine-B: 1–5%; phosphoric acid 10–30%; isopropanol 1–5%	1 phage (936 group) (25, 50 ppm) [28]
Iodine-C: 1–5%; phosphoric acid 10–30%; sodium iodide 1–5%; methyloxirane, polymer with oxirane 10–30%	1 phage (936 group) (12.5, 25 ppm) [28]
Iodine-D: 1–5%; phosphoric acid 10–30%; methyloxirane, polymer with oxirane, monobutyl ether 7–13%; dipropylene glycol monomethyl ether 1–5%	1 phage (936 group) (12.5, 25 ppm) [28]
**Amphoteric**	
Amphoteric: Alkylaminocarboxymethylaminopropane, sodium salt 5–10%	1 phage (936 group) (200, 500 ppm) [28]
Sodium hydroxide	11 phages (936 group) (0.2%) [22]
Lactic acid	11 phages (936 group) (300 mM) [22]
QAC: Alkyldimethyl benzyl ammonium chloride	11 phages (936 group) (0.1%) [22]
Virkon: 49.7% potassium monopersulphate, 49.7%	11 phages (936 group) (1%) [22]
Spor-Klenz: 4.5% peracetic acid, 22% hydrogen peroxide, and <10% acetic acid	11 phages (936 group) (pure) [22]
Benzalkonium chloride	36 phages (936 group) (0.08%) [5]
Polyvinylpyrrolidone-iodine	36 phages (936 group) (1%) [5]
Hydrogen peroxide	36 phages (936 group) (14%) [5]
Sodium percarbonate	36 phages (936 group) (1, 5, 10%) [5]
Sodium dichloroisocyanurate	36 phages (936 group) (5%, 10%, 20%) [5]
Sodium chlorite	36 phages (936 group) (1%, 5%, 10%, 20%) [5]
Sanitizer A: C8-C18 alkyldimethyl chloride ammonium compound	36 phages (936 group) (0.08%) [5]
Sanitizer B: Mixture containing 30–60% sodium hydroxide	36 phages (936 group) (0.1%) [5]
Sanitizer C: Mixture containing 5–10% sodium hydroxide, 2–5% sodium hypochlorite	36 phages (936 group) (0.12%) [5]
Sanitizer D: etanol 10%, chlorhexidine digluconate 10%, Tetradecyl-trimethyl-ammonium-bromide <1%	36 phages (936 group) (16%, 40%, 80%) [5]
Sanitizer E: Polymeric-biguanide-hydrochloride	36 phages (936 group) (0.1%, 1%, 5%, 10%) [5]
Sanitizer F: Mixture containing 30% nitric acid, 5% orthophosphoric acid	36 phages (936 group) (0.1%, 0.5%, 1%, 5%) [5]

**Table 4:** Thermal treatments (temperature, time, suspension medium) studied for inactivation of dairy phages.

Temperature	Number of Studied Phages (Treatment Time, Suspension Medium if Available)					
	*Streptococcus thermophilus*	*Leuconostoc mesenteroides (Lm)*/*pseudomesenteroides (Lp)*	*Lactobacillus helveticus*	*Lactobacillus delbrueckii*	*Lactobacillus casei/paracasei*	*Lactobacillus plantarum*
50–55 °C	1 phage (60 min) [44]	12 phages *Lp* (1 min, skim milk) [27]	1 phage (60 min) [44]	1 phage (60 min) [52]	1 phage (50 min, MRS broth) [38]	
	9 phages (1 min, UHT-skim milk [27]					
60–65 °C	1 phage (64 min, skim milk) [53]	5 phages *Lm* (45 min, TMG buffer, skim milk, MRS broth) [40]	1 phage (60 min) [44]	4 phages (45 min, TMG buffer, RSM, MRS broth) [32]	2 phages (45 min, TMG buffer, MRS broth, RSM) [33]	4 phages (45 min, MRS broth, RSM, EM medium, TMG buffer) [34]
	1 phage (60 min) [44]	1 phage *Lp* (45 min, TMG buffer, skim milk, MRS broth) [40]	4 phages (45 min, TMG buffer, RSM, MRS broth) [31]	2 phages (45 min, skim milk, MRS broth) [35]	2 phages (45 min, TMG buffer, MRS b roth, RSM) [43]	1 phage (60 min, MRS broth, RSM, TMG buffer) [37]
	5 phages (45 min, TMG buffer, ETS broth, RSM) [30]	66 phages *Lp* (1 min, UHT-skim milk, water) [49]		1 phage (60 min) [52]	1 phage (45 min, TMG buffer, MRS broth, RSM) [36]	1 phage (60 min, MRS broth, RSM, TMG buffer) [39]
	9 phages (1 min, UHT-skim milk [27]	11 phages *Lm* (1 min, UHT-skim milk, water) [49]		1 phage (50 min, MRS broth) [54]	1 phage (50 min, MRS broth) [38]	1 phage (60 min, water) [55]
	1 phage (90 min, water, skim milk) [27]	1 phage *Lp* (90 min, UHT-skim milk) [49]				
		12 phages *Lp* (1 min, UHT-skim milk) [27]				
		1 phage *Lp* (90 min, water, skim milk) [27]				
70–75 °C	1 phage (64 min, skim milk) [53]	5 phages *Lm* (45 min, TMG buffer, skim milk, MRS broth) [40]	1 phage (60 min) [44]	4 phages (45 min, TMG buffer, RSM, MRS broth) [32]	2 phages (45 min, TMG buffer, MRS broth, RSM) [33]	4 phages (45 min, MRS broth, RSM, EM medium, TMG buffer) [34]
	1 phage (60 min) [44]	1 phage *Lp* (45 min, TMG buffer, skim milk, MRS broth) [40]	4 phages (45 min, TMG buffer, RSM, MRS broth) [31]	2 phages (45 min, skim milk, MRS broth) [35]	2 phages (45 min, TMG buffer, MRS broth, RSM) [43]	1 phage (60 min, MRS broth, RSM, TMG buffer) [37]
	5 phages (45 min, TMG buffer, ETS broth, RSM) [30]	66 phages *Lp* (1 min, UHT-skim milk, water) [49]		1 phage (60 min) [52]	1 phage (45 min, TMG buffer, MRS broth, RSM) [36]	1 phage (60 min, MRS broth, RSM, TMG buffer) [39]
	9 phages (1 min, UHT-skim milk [27]	11 phages *Lm* (1 min, UHT-skim milk, water) [49]		1 phage (50 min, MRS broth) [54]	1 phage (50 min, MRS broth) [38]	1 phage (3 min, water) [56]
	1 phage (90 min, water, skim milk) [27]	1 phage *Lp* (90 min, UHT-skim milk) [49]				1 phage (60 min, water) [55]
		12 phages *Lp* (1 min, UHT-skim milk) [27]				
		1 phage *Lp* (90 min, water, skim milk, UF, WPI, MCC) [27]				
80–85 °C	9 phages (1 min, UHT-skim milk [27]	3 phages *Lm* (45 min, skim milk) [40]		2 phages (45 min, skim milk, MRS broth) [35]	1 phage (50 min, MRS broth) [38]	1 phage (3 min, water) [56]
	1 phage (90 min, water, skim milk) [27]	66 phages *Lp* (1 min, UHT-skim milk, water) [49]		1 phage (50 min, MRS broth) [54]		
		11 phages *Lm* (1 min, UHT-skim milk, water) [49]				
		1 phage *Lp* (90 min, UHT-skim milk) [49]				
		12 phages *Lp* (1 min, UHT-skim milk) [27]				
		1 phage *Lp* (90 min, water, skim milk) [27]				
90–100 °C	1 phage (60 min) [44]	5 phages *Lm* (45 min, TMG buffer, skim milk, MRS broth) [40]	1 phage (60 min) [44]	4 phages (45 min, TMG buffer, RSM, MRS broth) [32]	2 phages (45 min, TMG buffer, MRS broth, RSM) [33]	4 phages (45 min, MRS broth, RSM, EM medium, TMG buffer) [34]
	5 phages (45 min, TMG buffer, ETS broth, RSM) [30]	1 phage *Lp* (45 min, TMG buffer, skim milk, MRS broth) [40]	4 phages (45 min, TMG buffer, RSM, MRS broth) [31]	2 phages (45 min, skim milk, MRS broth) [35]	2 phages (45 min, TMG buffer, MRS broth, RSM) [43]	1 phage (60 min, MRS broth, RSM, TMG buffer) [37]
	4 phages (45 min, skim milk) [57]	1 phage *Lp* (90 min, water, skim milk) [27]		1 phage (50 min, MRS broth) [54]	1 phage (45 min, TMG buffer, MRS broth, RSM) [36]	1 phage (60 min, MRS broth, RSM, TMG buffer) [39]
	1 phage (90 min, water, skim milk) [27]				2 phages (30 min RSM, MRS broth, EM medium) [58]	1 phage (3 min, water) [56]
					8 phages (30 min RSM, MRS broth, EM medium) [59]	
					3 phages (45 min, skim milk) [57]	
